# Association of antinuclear antibody positivity with liver disease severity in pediatric metabolic dysfunction-associated steatotic liver disease

**DOI:** 10.3389/fped.2025.1527605

**Published:** 2025-02-26

**Authors:** Hyun Jin Kim, Ju Young Kim, Yoo Min Lee, Yong Hee Hong, Ben Kang, Byung-Ho Choe, Dae Yong Yi, Eun Hye Lee, Soon Chul Kim, You Jin Choi, Hyo-Jeong Jang, So Yoon Choi

**Affiliations:** ^1^ Department of Pediatrics, Chungnam National University Hospital, College of Medicine, Chungnam National University, Daejeon, Republic of Korea; ^2^ Department of Pediatrics, Daejeon Eulji Medical Center, Eulji University School of Medicine, Daejeon, Republic of Korea; ^3^ Department of Pediatrics, Soonchunhyang University Bucheon Hospital, Soonchunhyang University College of Medicine, Bucheon, Republic of Korea; ^4^ Department of Pediatrics, School of Medicine, Kyungpook National University, Daegu, Republic of Korea; ^5^ Department of Pediatrics, Chung-Ang University Hospital, College of Medicine, Chung-Ang University, Seoul, Republic of Korea; ^6^ Department of Pediatrics, Nowon Eulji Medical Center, Eulji University School of Medicine, Seoul, Republic of Korea; ^7^ Department of Pediatrics, Jeonbuk National University Medical School and Hospital, Jeonju, Republic of Korea; ^8^ Department of Pediatrics, Inje University, Ilsan Paik Hospital, Inje University College of Medicine, Ilsan, Republic of Korea; ^9^ Department of Pediatrics, Keimyung University School of Medicine, Daegu, Republic of Korea; ^10^ Department of Pediatrics, Kosin Gospel Hospital, Kosin University College of Medicine, Busan, Republic of Korea

**Keywords:** metabolic dysfunction-associated steatotic liver disease, antinuclear antibody, aspartate aminotransferase, alanine aminotransferase, pediatrics - children

## Abstract

**Background:**

Although antinuclear antibody (ANA) is frequently observed in patients with metabolic dysfunction-associated steatotic liver disease (MASLD), its clinical significance in children remains unclear and controversial. In this study, we investigated the prevalence of ANA positivity and the factors associated with it in pediatric MASLD patients without concurrent autoimmune hepatitis.

**Methods:**

We retrospectively reviewed the medical records of patients aged 4–18 years diagnosed with MASLD and tested for ANA from January 2015 to December 2020 at 10 hospitals in Korea. All statistical analyses were carried out using SPSS 26.0 and *P*-values <0.05 were considered statistically significant.

**Results:**

Out of the 439 patients included, ANAs were present in 89 (20.3%); 51 (57.3%) patients had ANA titer <1:80; 22 (24.7%), <1:160; 10 (11.2%), <1:320; and 6 (6.7%), <1:640. Compared to ANA-negative patients, aspartate aminotransferase (AST, *P* = 0.003) and alanine aminotransferase (ALT, *P* = 0.007) levels were significantly higher in ANA-positive patients. The ALT to Platelet Ratio Index (APRI) score was also associated with the ANA-positive patients (*P* = 0.005). To predict ANA positivity using APRI, the area under receiver operating characteristic (AUROC) curve was 0.597 (*p* = 0.004), and the APRI cutoff value of >0.893 could predict ANA, with sensitivity and specificity of 42.7% and 72.9%, respectively.

**Conclusions:**

ANA positivity in pediatric MASLD is associated with greater liver enzyme elevation and increased risk of fibrosis, highlighting the need for careful monitoring in ANA-positive patients.

## Background

Autoantibodies react with self-antigens and are directed against one or more of the individual's own proteins (1). Non-specific autoantibodies associated with liver disease include antinuclear, anti-smooth muscle, and anti-mitochondrial antibodies (2). Antinuclear and anti-smooth muscle antibodies are frequently positive in patients with autoimmune hepatitis (AIH), and their positivity is one of the diagnostic criteria for AIH, together with hypergammaglobulinemia and typical histological findings (3–5). However, low levels of these autoantibodies are also present in 6%–15% of the healthy population, highlighting their non-specific nature. In patients with chronic liver disease, 7%–52% have been reported to be positive for autoantibodies, as any component of hepatocytes can potentially trigger their production (6, 7).

Metabolic dysfunction-associated steatotic liver disease (MASLD) is diagnosed by exclusion through the presence of hepatic steatosis with no other causes (8, 9). In pediatric obese patients, alanine aminotransferase (ALT) measurement is currently the best screening tool for MASLD, though it has significant limitations. While liver biopsy remains the gold standard for diagnosing MASLD, it is challenging to perform in children due to its invasive nature, need for sedation, and potential complications such as pain, bleeding, and, rarely, mortality (10). Consequently, pediatric MASLD is commonly diagnosed using clinical symptoms, laboratory findings, and imaging rather than biopsy.

Autoantibody testing is often recommended when MASLD is clinically suspected to rule out other potential causes. However, the prevalence and clinical significance of autoantibodies in MASLD, particularly in pediatric patients, are not well established, and studies on ANA in this population are especially limited. In this study, we aimed to investigate the prevalence of antinuclear antibodies and their association with the degree of steatosis and fibrosis in pediatric patients with MASLD.

## Methods

### Patients and study design

This was a retrospective multicenter study in the pediatric departments of 10 hospitals in Korea: Chungnam National University Hospital, Chung-Ang University Hospital, Jeonbuk National University Hospital, Kyungpook National University Children's Hospital, Soonchunhyang University Bucheon Hospital, Nowon Eulji Medical Center, Daejeon Eulji Medical Center, Keimyung University Dongsan Medical Center, Inje University Ilsan Paik Hospital, and Kosin University Gospel Hospital.

Among patients aged 4–18 years who were diagnosed with MASLD between January 2015 and December 2020, we included only those who had undergone ANA testing at the time of diagnosis. Patients who were diagnosed with autoimmune hepatitis or had other chronic liver diseases were excluded.

Baseline clinical patients' data, such as sex, age, height, weight, and body mass index (BMI), were collected using electronic medical records. Laboratory tests included tests for levels of ALT, aspartate aminotransferase (AST), gamma-glutamyl transferase, total cholesterol, triglyceride, low-density lipoprotein and high-density lipoprotein (HDL) cholesterol, and fasting glucose. ANA tests were performed via indirect immunofluorescence on Hep-2 cells. The ALT to Platelet Ratio Index (APRI) score for noninvasive markers of liver fibrosis was calculated as follows: APRI score = AST level (IU/L)/AST upper limit of normal (IU/L)/platelet count (10^9^/L) (11, 12).

MASLD was diagnosed based on bright or hyperechoic lesions on liver imaging and ALT levels ≥30 IU/L (8). ANA-positivity was defined as ANA titer of ≥1:80 since the detection of low ANA titer is evident even in the healthy population (13). Ultrasonographic evaluation for the diagnosis of fatty liver was conducted by experienced pediatric radiologists who were blinded to the patients' clinical and laboratory data. The diagnosis of hepatic steatosis was based on specific sonographic features, including increased liver parenchymal echogenicity (bright liver) relative to the adjacent kidney and spleen, absence of focal hepatic lesions, enhanced posterior beam attenuation, and reduced clarity of the portal and hepatic vein structures. The severity of hepatic steatosis was graded semiquantitatively as mild (grade 1), moderate (grade 2), or severe (grade 3), following the criteria described by Saadeh et al. (14–16) This assessment inherently carries operator dependency, and neither the hepatorenal index nor artificial intelligence-based image processing techniques were employed in this study. Diabetes mellitus was declared when the fasting plasma glucose level was ≥126 mg/dl or a 2-h oral glucose tolerance test result was ≥200 mg/dl (17, 18). Hypertension was defined as repeated blood pressure values greater than the 95th percentile for the age, sex, and height of that patient at three separate visits (19, 20).

For detecting cirrhosis, using an APRI cutoff score of 2.0 was more specific (91%) but less sensitive (46%). APRI scores of ≤0.3 and ≤0.5 ruled out significant fibrosis and cirrhosis, respectively, and a value of ≥1.5 ruled out significant fibrosis (12, 21).

### Ethics approval and consent to participate

This study was approved by the Institutional Review Board (IRB) of Chungnam National University Hospital and all other participating centers (IRB number 2019-11-029). This study was conducted according to the guidelines laid down in the Declaration of Helsinki. The need for Informed Consent was waived by the IRB of Chungnam National University Hospital due to the retrospective nature of the study.

### Statistical analysis

Variables were summarized by frequency and percentage for categorical data and mean ± standard deviation for numeric data. Group differences were tested using the chi-squared test or Fisher's exact for categorical data and independent *t*-test or Mann–Whitney *U*-test and analysis of variance or Kruskal–Wallis test for numeric data as appropriate. To check if its distribution is normal, we used Shapiro–Wilk's test. Univariate and multivariate logistic regression analysis were performed to identify prognostic factors which are independently related to ANA. The receiver operating characteristic (ROC) curve analysis was used to calculate the area under the curve (AUC) and performed to assess the sensitivity and specificity of APRI for predicting ANA. The cutoff value was determined by Youden's index. All statistical analyses were carried out using SPSS 26.0 statistical software (IBM Corp. Released 2019. IBM SPSS Statistics for Windows, Version 26.0. Armonk, NY: IBM Corp.) and MedCalc Statistical Software version 19.2.6 (MedCalc Software Ltd., Ostend, Belgium). Statistical consultation for the analyses was performed by ACE Statistical Consulting in the Republic of Korea. *P*-values less than 0.05 was considered statistically significant.

## Results

### Comparison of patients’ baseline characteristics in ANA-positive and -negative groups

**Figure 1:**
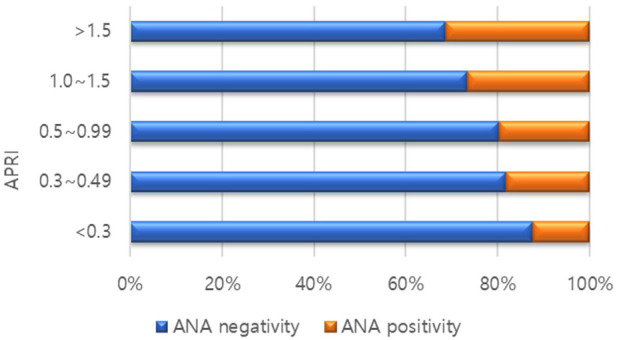
Proportion of antinuclear antibody (ANA) positivity according to ALT to platelet ratio Index (APRI) scores.

A total of 439 patients were included in the study; 89 (20.3%) were ANA-positive, and 350 (79.7%) were ANA-negative. A comparison of the baseline characteristics of the ANA-positive and ANA-negative groups is presented in Table 1. AST (94.60 ± 91.03 IU/L vs. 72.36 ± 50.22 IU/L, *P* = 0.009) and ALT (155.37 IU/L ± 96.16 vs. 125.70 ± 82.60 IU/L, *P* = 0.007) levels were significantly higher in the ANA-positive patient group than in the ANA-negative patient group (Table 2). There was no difference in the degree of steatosis between the two groups, as confirmed by ultrasonography. However, APRI, an indirect indicator of fibrosis, was significantly higher in the ANA-positive group (0.91 ± 0.63 vs. 0.73 ± 0.56, *P* = 0.005). In addition, the higher the APRI value, the higher the proportion of ANA-positive patients (Figure 1).

**Table 1:** Patient characteristics.

Variable	Overall (*n* = 439)
Age (years)	12.45 ± 3.08
Sex, male, *n* (%)	331 (75.4)
BMI *z*-score	2.05 ± 1.04
ALT (IU/L)	131.71 ± 86.24
AST (IU/L)	69.20 ± 49.50
Total cholesterol (mg/dl)	183.00 ± 36.61
Triglyceride (mg/dl)	156.39 ± 81.93
HDL-cholesterol (mg/dl)	46.17 ± 9.92
LDL-cholesterol (mg/dl)	118.11 ± 33.29
Fasting Glucose (mg/dl)	100.76 ± 29.37
Diabetes mellitus, *n* (%)	22 (5.0)
Hypertension, *n* (%)	22 (5.0)
Liver U/S grade, *n* (%)	
Mild	156 (35.5)
Moderate	215 (49.0)
Severe	68 (15.5)
APRI score	0.77 ± 0.58

ANA, antinuclear antibody; AST, aspartate aminotransferase; ALT, alanine aminotransferase; APRI, ALT to platelet ratio index, BMI, body mass index; HDL, high-density lipoprotein; LDL, low-density lipoprotein; U/S, ultrasound.

Data are presented as mean ± SD or number (%), unless otherwise indicated.

Shapiro–Wilk's test was employed for test of normality assumption.

**Table 2:** Comparison of patients’ baseline characteristics in ANA positive and ANA negative groups.

Variable	Group		*P*-value
	ANA (+)	ANA (−)	
*N* (%)	89 (20.3)	350 (79.7)	
Age (years)	12.01 ± 2.53	12.57 ± 3.20	0.051**
Sex, male, *n* (%)	67 (75.3)	266 (75.4)	0.977***
BMI *z*-score	2.27 ± 3.00	2.00 ± 0.58	0.312**
ALT (IU/L)	155.37 ± 96.16	125.70 ± 82.60	0.007**
AST (IU/L)	80.48 ± 48.46	66.33 ± 49.42	0.003**
Total cholesterol (mg/dl)	183.53 ± 37.20	182.86 ± 36.51	0.879*
Triglyceride (mg/dl)	145.63 ± 62.61	159.23 ± 86.18	0.464**
HDL-cholesterol (mg/dl)	46.70 ± 7.84	46.05 ± 10.38	0.226**
LDL-cholesterol (mg/dl)	119.74 ± 38.48	117.68 ± 31.85	0.648*
Fasting Glucose (mg/dl)	98.15 ± 18.18	101.41 ± 31.56	0.541**
Diabetes mellitus, *n* (%)	4 (4.5)	18 (5.1)	1.000****
Hypertension, *n* (%)	5 (5.6)	17 (4.8)	0.786****
Liver U/S grade, *n* (%)			
Mild	30 (33.7)	128 (36.3)	0.324***
Moderate	49 (55.1)	166 (47.0)	
Severe	10 (11.2)	59 (16.7)	
APRI score	0.91 ± 0.63	0.73 ± 0.56	0.005**

ANA, antinuclear antibody; AST, aspartate aminotransferase; ALT, alanine aminotransferase; APRI, ALT to platelet ratio index, BMI, body mass index; HDL, high-density lipoprotein; LDL, low-density lipoprotein; U/S, ultrasound.

Data are presented as mean ± SD or number (%), unless otherwise indicated.

Shapiro–Wilk's test was employed for test of normality assumption.

* *P*-values were derived from independent *t*-test.

** *P*-values were derived from Mann–Whitney's *U*-test.

*** *P*-values were derived from chi-square test.

**** *P*-values were derived from Fisher's exact test.

### Risk factor analysis for ANA

Risk factor analysis for ANA is shown in Table 3. In the univariate analysis, ALT [odds ratio (OR) 1.00, 95% confidence interval (CI): 1.00–1.01, *P* = .004], AST [OR 1.01, 95% CI: 1.00–1.01, *P* = .019], and APRI score [OR 1.62, 95% CI: 1.12–2.34, *P* = .011] were related to ANA although only ALT [OR 1.00, 95% CI: 1.00–1.01, *P* = .004] was related to ANA in the multivariate analysis.

**Table 3:** Risk factor analysis for positivity of anti-nuclear antibody.

	Univariate analysis			Multivariate analysis		
Variable	OR	95% CI	*P*	OR	95% CI	*P*
Age (years)	0.94	(0.87–1.02)	0.128			
Sex, male, *n* (%)	1.01	(0.59–1.73)	0.977			
BMI *z*-score	0.96	(0.92–1.00)	0.189			
ALT (IU/L)	1.00	(1.00–1.01)	0.004	1.00	(1.00–1.01)	.004
AST (IU/L)	1.01	(1.00–1.01)	0.019			
Total cholesterol (mg/dl)	1.00	(0.99–1.01)	0.879			
Triglyceride (mg/dl)	1.00	(0.99–1.00)	0.191			
HDL-cholesterol (mg/dl)	1.01	(0.98–1.03)	0.626			
LDL-cholesterol (mg/dl)	1.00	(0.99–1.01)	0.647			
Fasting Glucose (mg/dl)	1.00	(0.99–1.01)	0.366			
Diabetes mellitus, *n* (%)	0.87	(0.29–2.63)	0.802			
Hypertension, *n* (%)	1.17	(0.42–3.25)	0.769			
Liver U/S grade, *n* (%)						
Mild	1.00	–	–			
Moderate	1.24	(0.74–2.06)	0.409			
Severe	0.72	(0.33–1.58)	0.418			
APRI score	1.62	(1.12–2.34)	0.011			

The effect of independent variables on ANA was analyzed using the multivariate logistic regression, and the statistically significant variables were included in the univariate logistic regression with 0.05 alpha level. The multivariate model was created using a backward elimination method, and the probability was set at 0.05 for elimination. The mild liver U/S grade was used as the reference category in the logistic regression analysis. Therefore, the OR is set to 1.00, and corresponding 95% confidence intervals and *p*-values are not applicable.

### Receiver operating characteristic (ROC) curves of APRI to predict positivity of ANA

**Figure 2:**
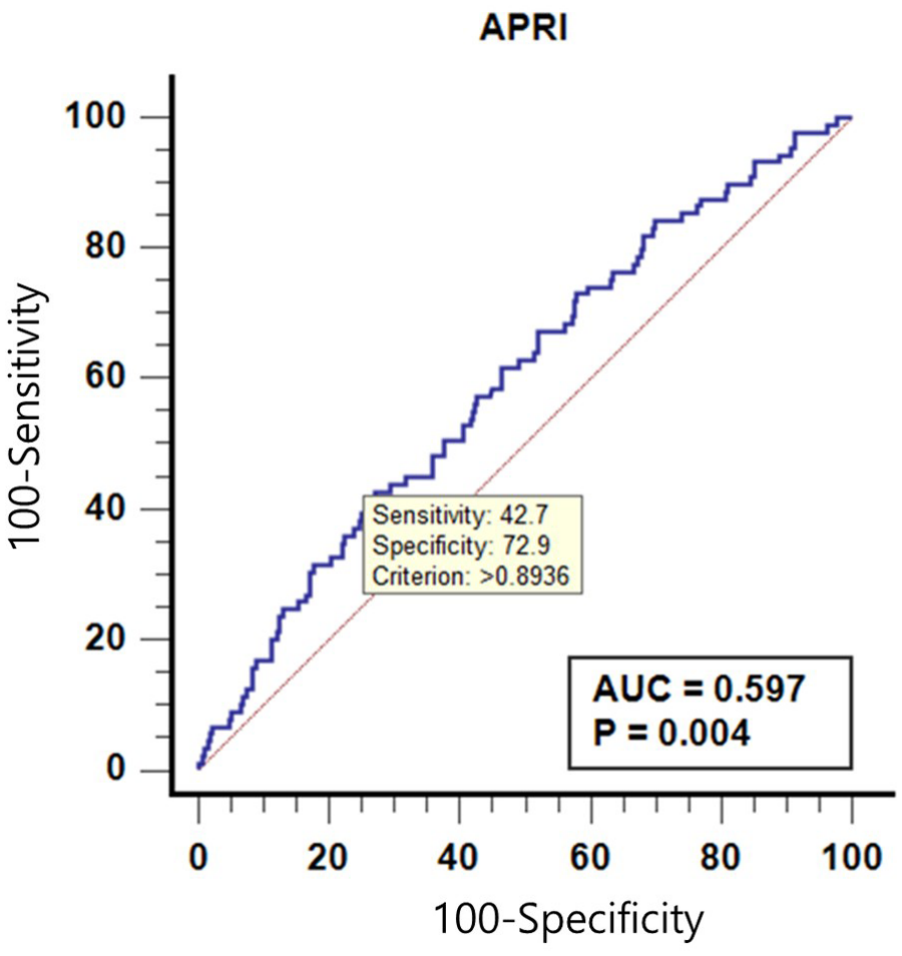
Receiver operating characteristic curves of alanine aminotransferase to platelet ratio Index (APRI) to predict anti-nuclear antibody (ANA).

A receiver operating characteristic (ROC) curves of APRI to predict ANA is shown in Table 4 and Figure 2. The area under receiver operating characteristic (AUROC) curve was 0.597 (*p* = 0.004), and the APRI cutoff value of >0.893 could predict positive-ANA, with sensitivity and specificity of 42.7% and 72.9%, respectively.

**Table 4:** Receiver operating characteristic curve analysis for predicting positive anti-nuclear antibody.

Variable	Cut-point value	Group		Cut-point value	AUC (*p*)	Sensitivity, %	Specificity, %	PPV, %	NPV, %
		ANA (+)	ANA (−)						
APRI score	>0.893	38	95	>0.893	0.597 (0.004)	42.7	72.9	28.6	83.3
	≤0.893	51	255						
Total		89	350						

Sensitivity: 38/89 × 100% = 42.7%.

Specificity: 255/350 × 100% = 72.9%.

False negative rate (100%-sensitivity) = 51/89 × 100% = 57.3%.

False positive rate (100%-specificity) = 95/350 × 100% = 27.1%.

Positive predicted value = 38/133 × 100% = 28.6%.

Negative predicted value = 255/306 × 100% = 83.3%.

Concordance between group regarding to APRI and ANA (+) and ANA (-) was good (293/439 × 100%=66.7%).

## Discussion

In this study, we found that 20.3% of pediatric patients with MASLD tested positive for ANA, with varying titers observed across the cohort. Notably, ANA-positive patients exhibited significantly higher levels of AST and ALT compared to ANA-negative patients, suggesting that ANA positivity is associated with greater liver inflammation. However, there was no significant difference in the degree of hepatic steatosis between the two groups as assessed by ultrasonography. Additionally, the APRI score, a non-invasive index commonly used to assess liver fibrosis, was significantly elevated in ANA-positive patients, supporting a potential link between ANA positivity and increased fibrosis risk in pediatric MASLD.

Interestingly, in our multivariate analysis, only ALT remained significantly associated with ANA positivity, while other factors, including AST and APRI, did not retain statistical significance after adjustment. This may be explained by the fact that ALT is a more specific marker of hepatocellular injury compared to AST, which is also found in other tissues, including muscle and the heart (22). The stronger association of ALT with ANA positivity may reflect its closer link to ongoing liver inflammation specifically related to MASLD, whereas AST elevations may be influenced by extrahepatic factors. Additionally, ALT elevation may indicate subclinical immune-mediated hepatocellular injury that could be linked to autoimmune responses reflected by ANA positivity (23). The absence of significant associations with other variables suggests that ALT may serve as a more sensitive marker of immune-related liver injury in this population.

The association between ANA positivity and MASLD may be partially explained by insulin resistance. Previous studies have reported a close link between high-titer ANA positivity and elevated indices of insulin resistance, a well-known factor in MASLD pathogenesis (24, 25). Hepatic NKT cell accumulation, which promotes fibrosis in liver disease, can also produce autoantibodies in MASLD (26). However, the clinical significance of ANA in patients with MASLD is conflicting and controversial. Yodoshi et al. found a strong association between positive ANA and higher steatosis scores, while Adams et al. demonstrated that ANA-positive NASH patients had more severe liver necroinflammation and fibrosis than ANA-negative patients (27, 28). In contrast, Kohut et al. observed no association between autoantibodies and the degree of liver inflammation, steatosis, or fibrosis, though they noted that combined ALT and ANA positivity could improve identification of patients at higher risk for NASH (29). In our study, while there was no difference in the degree of steatosis between ANA-positive and ANA-negative groups, ALT and APRI levels were significantly higher in ANA-positive patients.

The APRI's predictive value for ANA positivity was modest, with an AUROC curve of 0.597. An APRI cutoff of >0.893 showed a sensitivity of 42.7% and specificity of 72.9% for predicting ANA positivity. While these values indicate limited utility for APRI in reliably identifying ANA-positive patients, they suggest that higher APRI scores might warrant closer monitoring of pediatric MASLD patients, especially those with elevated liver enzymes.

Additionally, previous studies have suggested that MASLD may worsen clinical outcomes in patients with AIH. Johnson et al. reported that patients with combined AIH and NASH experienced poorer survival and more adverse outcomes than those with AIH alone, underscoring the need for caution when ANA is positive in patients with MASLD or concurrent AIH (30).

Long-term studies of ANA-positive MASLD patients, especially in children, remain limited. One recent study noted that ANA-positive MASLD patients had a higher prevalence of NASH at diagnosis; however, long-term outcomes, including hepatocellular carcinoma occurrence, extrahepatic malignancy, and overall survival, were similar to those of ANA-negative patients (31). In our study, a high ANA titer (1:320) was not associated with significant fibrosis in MASLD patients, nor was there a significant elevation in AST, ALT, or APRI levels in patients with higher ANA titers. This aligns with previous findings that significant ANA positivity (ANA ≥1:160) is not necessarily linked to advanced histological features in MASLD (32). Interestingly, the APRI cutoff value for predicting ANA positivity (0.893) in our study was higher than the optimal APRI score of 0.64, typically used to predict advanced fibrosis (F3/F4) in chronic hepatitis C patients (33).

This study had several limitations. First, its retrospective design may have affected the consistency of some variables. Second, the operator-dependent nature of ultrasonography in diagnosing hepatic steatosis is a notable limitation. Since this was a multicenter study, sonographic assessments were conducted by multiple radiologists, which may have introduced inter-operator variability and influenced the evaluation of hepatic steatosis severity. Recent studies have shown artificial intelligence-based ultrasonographic algorithms can automatically calculate the hepatorenal index, significantly improving the diagnostic performance for mild hepatic steatosis and reducing operator dependency (34). Such AI-driven tools are expected to contribute to more accurate and standardized assessments of hepatic steatosis in the future. While ALT levels were analyzed in relation to ANA, they were not adjusted for BMI, which represents a limitation of our study. Despite these limitations, our study is valuable in that it evaluates the significance of ANA positivity in a relatively large cohort of pediatric MASLD patients.

In conclusion, ANA positivity in pediatric MASLD is found to be associated with elevated liver enzymes and increased fibrosis risk, as indicated by higher APRI scores in ANA-positive patients. These results underscore the need for careful monitoring and potentially more aggressive management strategies in ANA-positive children with MASLD. Future research should aim to further elucidate the role of ANA in pediatric MASLD pathogenesis and assess its utility as a biomarker for disease severity and progression.

## Data Availability

The raw data supporting the conclusions of this article will be made available by the authors, without undue reservation.
